# Validation of the Accuracy and Convergence Time of Real Time Kinematic Results Using a Single Galileo Navigation System

**DOI:** 10.3390/s18082412

**Published:** 2018-07-25

**Authors:** Zbigniew Siejka

**Affiliations:** Department of Geodesy, University of Agriculture in Krakow, Al. Mickiewicza 21, 31-120 Krakow, Poland; rmsiejka@cyf-kr.edu.pl; Tel.: +48-12-662-4539

**Keywords:** GPS, Galileo, Multi-GNSS precise positioning, convergence time

## Abstract

For the last two decades, the American GPS and Russian GLONASS were the basic systems used in global positioning and navigation. In recent years, there has been significant progress in the development of positioning systems. New regional systems have been created, i.e., the Japanese Quasi-Zenith Satellite System (QZSS) and Indian Regional Navigational Satellite System (IRNSS). A plan to build its own regional navigation system named Korean Positioning System (KPS) was announced South Korea on 5 February 2018. Currently, two new global navigation systems are under development: the European Galileo and the Chinese BeiDou. The full operability of both systems by 2020 is planned. The paper deals with a possibility of determination of the user’s position from individual and independent global navigation satellite system (GNSS). The article is a broader concept aimed at independent determination of precise position from individual GPS, GLONASS, BeiDou and Galileo systems. It presents real time positioning results (Real Time Kinematic-RTK) using signals from Galileo satellites only. During the test, 14 Galileo satellites were used and the number of simultaneously observed Galileo satellites varied from five to seven. Real-time measurements were only possible in certain 24-h observation windows. However, their number was completed within 6 days at the end of 2017 and beginning of 2018, so there was possible to infer about the current availability, continuity, convergence time and accuracy of the RTK measurements. In addition, the systematic errors were demonstrated for the Galileo system.

## 1. Introduction

For a long time, satellite navigation and positioning relied mainly on the American GPS. Later, the Russian GLONASS was used as an additional system. Currently, thanks to the two new Galileo and BeiDou, positioning without GPS is possible. The Galileo system has already passed the initial phase of orbital validation (In-orbit Validation-IOV), which included testing and checking of four operational satellites and related terrestrial infrastructure. Currently, Galileo is in the reaching of Full Operational Capability (FOC) phase. The completed Galileo constellation will consist of 30 satellites located on the Middle Earth Orbits (MEO) [1]. The constellation of 18 Galileo satellites at the end of 2016, and then thanks to the provision of precise products in the real time by IGS (International GNSS Service) Real Time (RT) measurements have become possible. These measurements are important not only for surveyors, but also for many communities, including those dealing with geographic information systems, precise navigation and many others. It is the first civilian global positioning system, expected for a long time not only in Europe but also in other parts of the world. It uses experience of its predecessors: American GPS and Russian GLONASS and therefore has several important advantages over them. Galileo uses, among others, a new more accurate model of ionosphere (NeQuick) and instead of one signal in one frequency, it generates signals in two frequencies called data and pilot. Data–includes navigational messages. Pilot–is used to determine the pseudoranges. In addition, all Galileo satellites are equipped with retroreflectors to track them by satellite laser ranging (SLR), which allows the assessment of the quality of the orbit in space.

So far, we find in papers the results of research, which refer to the use of newly built navigation systems Galileo and BeiDou in combination with GPS and GLONASS. Research in this area has been published by many scientists from around the world, they relate to precise GNSS positioning in real time or in postprocessing [2,3,4,5,6]. There are also research projects in this area, such as Multi-GNSS Experiment (MGEX) [7,8]. Another direction is the integrated, compatible GNSS used in precise point positioning in real time using corrections provided by satellite from large-scale satellite networks [9,10,11].

In this paper, precise relative positioning, performed by RTK method based only on signals from Galileo satellites is carried out. The research was carried out in three main aspects. The first included determining the availability and continuity of positioning. The second concerned the convergence time as the speed of obtaining a precise position with given accuracy. The third aspect included research on determining the real accuracy of relative positioning in real time. The research was carried out using signals of Galileo satellites, from December 2017–January 2018.

## 2. Materials

### 2.1. Assessment of Availability and Continuity of Positioning Based on the Constellation of Galileo Satellites

**Figure 1:**
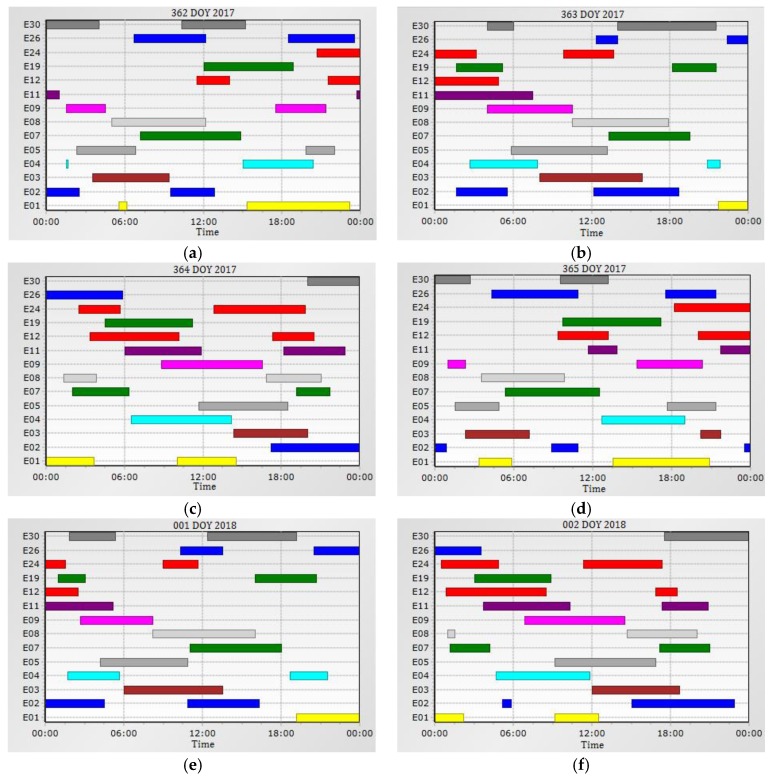
Visibility of the operational satellites of the Galileo constellation at the site Kraków–Poland; (**a**) 362 day of year 2017; (**b**) 363 day of year 2017; (**c**) 364 day of year 2017; (**d**) 365 day of year 2017; (**e**) 1 day of year 2018; (**f**) 2 day of year 2018. Source: Own elaboration based on “GNSS Planning Online”.

The constellation of the Galileo satellites is not yet complete, and FOC is expected in 2020. For this reason, RTK test measurements were therefore only possible to be made in specific time windows within 24 h, when the number of observed satellites was at least 5 or more. In the site, Krakow-Poland (φ = 50.0207, λ = 20.0221), during time span (362–365 DOY 2017 and 001–002 DOY 2018) it was possible to observe simultaneously from one to seven satellites on the elevation above 10° (Figure 1). Current (December 2017) the constellation of Galileo satellites, consists of 18 satellites, including 14 fully operational ones. Four satellites of the Galileo constellation are indicated as unusable “Unh–Unhealthy)” for various reasons. Two satellites: E14, Galileo 5 (Galileo-FOC FM1, GSAT 0201, Doresa) and E18, Galileo 6 (Galileo-FOC2, GSAT 0202, Milena) launched in August 2014, due to the failure of the Russian Soyuz-ST-B Fregat-MT carrier rocket, were placed on an elliptical orbit with too high eccentricity. Therefore, they cannot be used for navigation purposes nor positioning. Moreover, the satellite E20, Galileo-IOV FM4 (GSAT0104, Sif), broadcast only 1 frequency (E1-1575.42 MHz) due to power failure which occured on 27 May 2016. It also does not send a navigational message, therefore it can not be used in precise real-time positioning (GPS World-August 2017). The satellite E22, Galileo 8 (Galileo-FOC FM4, GSAT 0204, Anastasia), from 08 December 2017 is not available. It was removed until cancellation from the active constellation, due to problems with the on-board clock.

The orbits of Galileo operational satellites are located at an altitude of approximately 23.222 thousand km above the equator, which means that, on average, Galileo satellites have higher orbits by more than 3000 km relative to GPS satellites. The angle of inclination of the planes of Galileo satellites relative to the equator plane is greater by approximately 1° compared to the satellites of the GPS constellation. The eccentricities of the orbits of the Galileo satellites are in the range of E07 = 0.000080 to E24 = 0.000358.

Table 1, Table 2, Table 3, Table 4, Table 5 and Table 6 contain the following data: identified time intervals, the number of available Galileo satellites and visibility times of individual satellite constellations in the considered period, i.e., from 28 December 2017 to 2 January 2018. In addition, a detailed list of satellites is provided for time intervals in which at least five satellites were available above 10° horizontal angle. The performed research shows that 62 time intervals were recorded with a duration from 10 min to 1 h 50 min with sufficient number of satellites for RTK measurements (5–7).

The research carried out (Table 7) showed that the constellation of Galileo satellites allowed measurements for 18.75–49.31% in the following observation days.

The problem of discontinuity in observations occurs for kinematic and static measurements. It appears when a new satellite constellation is being built until the full operational capability is completed. Discontinuities in observations also occur when using fully operational systems such as GPS and GLONASS but measurements are made under difficult observational conditions for example, in wooded areas or places where there are other obstacles limiting access to satellites. Such situations occur usually in the case of rapid static measurements [12] or measurements in urban canyons [13]. The discontinuity problem occur also in the case of Precise Point Positioning (PPP) using different GNSS [14]. The references considering discontinuities in satellite observations proposed by the reviewer were added as a source of additional valuable information.

### 2.2. Study of the GNSS Receiver Convergence Time for a Specified Accuracy of the RTK Solution

The study involved the determination of the convergence time of the Trimble R10 receiver, in RTK measurements, for a single baseline <14 km, for the accuracy of horizontal and vertical measurements ≤0.05 m using only Galileo satellites [15]. The convergence time included the RTK measurement initialization time, which was counted from the moment the mobile receiver “rover” was connected to the assistance system (correction from KRUR—single reference station) until the assumed accuracy of the solution was achieved (≤0.05 m). Convergence time varies depending on the state of Galileo constellation, the level of multithreading, the proximity of obstacles such as high trees and buildings, and the calculation algorithms used by the receiver. The software used for experimental measurements of the Trimble R10 receiver, includes the new processing algorithm—Trimble HD-GNSS. It can use all GNSS constellations and implements a new approach in determination of the phase uncertainty. The applied solution determines the total number of phase cycles in a continuous manner based on data from subsequent measurement epochs [16]. It omits the traditional, two-stage method of initiating RTK measurements with taking into account the transition from the “float” solution to the “fixed” one.

Table 8 summarizes the detailed results of the obtained convergence times. During the tests, they ranged from 10 s to 10 min and 10 s. The shortest convergence times ≤10 s occurred in 14 cases, with the most advantageous satellites positions at the observation site, when PDOP (Position Dilution of Precision) ˂3.0. The initialization time from the interval (20 s–59 s) occurred in 5 cases when the PDOP values were within the range (3.26–4.81). Convergence times (1 min 20 s–4 min 00 s) occurred in 5 cases when the PDOP values were within the range (3.04–6.01). The longest convergence times in the interval (5 min 20 s–10 min 10 s) occurred in the remaining 6 cases when the PDOP values were within the range (2.82–6.21). In general, it can be concluded that the initial value of the PDOP parameter determines the duration of the convergence strict (in the case under study accuracy ≤0.05 m was arbitrarily assumed). However, this is not a strict rule. As can be seen from Table 8, in some cases lower values (theoretically more favorable) of the PDOP yielded longer initialization times. This confirms the well-known principle that the PDOP is only one of several parameters which is correlated with the duration of the initialization time. The results obtained indicate that it is also important which satellites are observed at a given moment. Each navigation satellite is characterized by its unique observational parameters, which in general can be described as non-system equipment noise of satellites. Unfortunately Trimble R10 GNSS receiver software (Trimble Access TM), used to manage measurements in real time, does not allow to obtain detailed information about satellites participating in a given solution. However, there is no information about the names of satellites. Therefore, a detailed analysis of the data in this respect is hindered.

### 2.3. Accuracy of Geodetic, Precise Positioning with the Use of RTK Method Based on Observations of Galileo Satellites

The main purpose of this work is to present real capabilities and performance of precise, geodetic GNSS real time measurements, using signals from Galileo satellites only and corrections from ground reference stations. The constellation of 14 fully operational Galileo satellites now enables RTK (Real Time Kinematic) measurements. However, it is possible only in certain time spans during a day and in specific areas of the globe. These capabilities were used to analyze the availability of Galileo satellites, first in general for the territory of Poland, and then for the selected site (Kraków φ = 50.0207, λ = 20.0221). Measurements were made within six days to cover a sufficiently long period of time and thus give reliable results. The period 362–365 DOY 2017 (GPS WEEK 1981) and 001–002 DOY 2018 (GPS WEEK 1982) was selected. On the basis of detailed assessments of availability and continuity of positioning carried out for the Galileo system (Table 1, Table 2, Table 3, Table 4, Table 5, Table 6 and Table 7) and pre-identified 62 time intervals in which five or more satellites were available, 36 time windows were selected with a duration of 30 min to 1 h.

**Figure 2:**
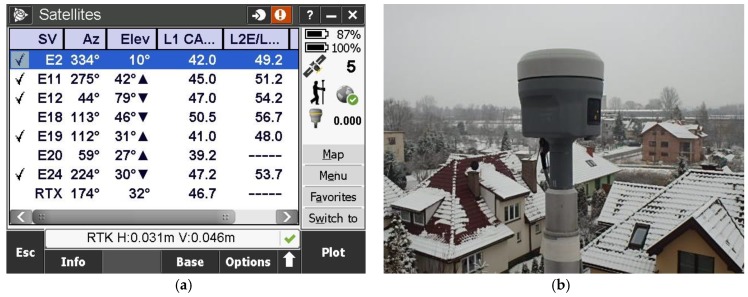
(**a**) Static measuring point, Trimble R10 antenna; (**b**) Measurement screen of the TSC3 controller.

**Figure 3:**
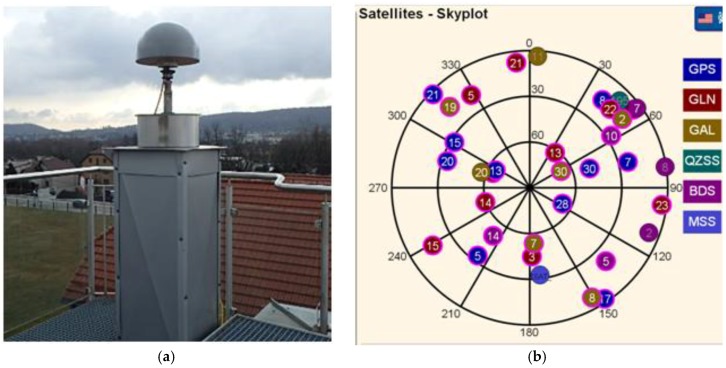
View of: (**a**) Reference station KRUR; (**b**) Skyplot satellites KRUR.

In the measurements the Trimble R10 company kit with the TSC3 controller were used (Figure 2). The Trimble R10, is a new generation, 440 channel integrated with the R10 Internal antenna, multi-system satellite receiver. It can simultaneously track up to 18 observation codes. It is adapted for tracking all currently available positioning and navigation systems: GPS (L1-C/A, L2E, L2C, L5), GLONASS (L1-C/A, L1P, L2C/A, L3), Galileo (E1, E5-A, E5-B, E5AltBOC), BeiDou (B1, B2), QZSS (L1-C/A, L1-SAIF, L1C, L2C, L5), SBAS (L1-C/A, L5), MSS (Service: RTX/xFill) (Trimble AccessTM software 2015). RTK corrections were provided by the KRUR scientific reference station, (Figure 3) working permanently since 1 November 2009 at the University of Agriculture in Krakow. The GNSS receiving antenna is located on a special observation pillar built for this purpose integrated into the building of the Faculty of Environmental Engineering and Geodesy at Balice 253C Street in Krakow. The observation pillar is independent of the entire building. Its upper part reaches about 1.5 m above the roof of the building (Figure 3), so that the satellite antenna installed on the pillar has a fully open observation horizon.

The reference station KRUR works on the basis of the Trimble NetR9 measurement set (Serial Number: 5025K68510) and antenna GNSS Choke w/SCIS Dome (Serial Number: 5511356093) with the individual calibration. This station observes and provides differential corrections to all currently available navigation systems (Figure 3b: GPS, GLONASS, Galileo, BeiDou QZSS). For the purpose of research carried out as a part of this work, multiple RTK measurements were made using the KRUR station as a single reference station. During experimental measurements, the KRUR station provided corrections to the observed satellites of the Galileo system via a mobile internet connection.

The KRUR reference station determines satellite corrections based on the internal Trimble NetR9 receiver software—Active Firmware Version 5.30. During 36 completed observation sessions more than 9700 of repetitive coordinate determinations at a static checkpoint were made. For each measurement, the following information was recorded in the automatic mode with an interval of 10 s, in accordance with:

- the name of the base point
- the name of the measuring point
- solution type
- date and time of measurement
- the height of the antenna
- components of vectors: ECEF: ∆X, ∆Y, ∆Z related to the base reference station
- the value of the PDOP parameter at which the position was determined
- the total number of tracked satellites, on the basis of which the position was determined
- detailed list of satellites of particular navigation systems (GPS, GLO, GAL, BDS) that were involved in the position determination
- coordinates (*x*-north, *y*-east, *h*-height) in the Polska/CS2000 system
- horizontal and vertical precision obtained when determining subsequent positions.

In the experimental measurements, the Real Time Kinematic (RTK) measurement technique was used with On-The-Fly initialization (OTF), which allows the position to be determined in the “near” real time with the centimeter accuracy. The OTF initialization consists in a quick solution of the problem of the uncertainty of phase measurements by a mobile receiver (rover) on the basis of data telemetrically transmitted from the reference station corrections to the pseudoranges and raw data for the phase measurements. The measurements used a single KRUR reference station, which carried out continuous observations and sent corrections in the CMRx format via mobile Internet to the rover receiver. The paper includes only measurement results, in which a full solution of the ambiguity of measurements by the rover receiver was obtained.

Time delays in providing correction data from reference stations to a real-time rover receiver are generally unavoidable. The size of this delay affects the accuracy of the position being determined. As demonstrated in the work [17] in the Real-time differential positioning (RTD) simultaneous use of several GNSS systems can compensate for longer time delays of delivered corrections in relation to positioning with the use of only one positioning system (e.g., GPS or BeiDou) while maintaining the same positioning accuracy. In the present paper, it is assumed that the magnitude of this delay cannot be greater than 1 s due to the fact that the positioning used the single Galileo system.

**Figure 4:**
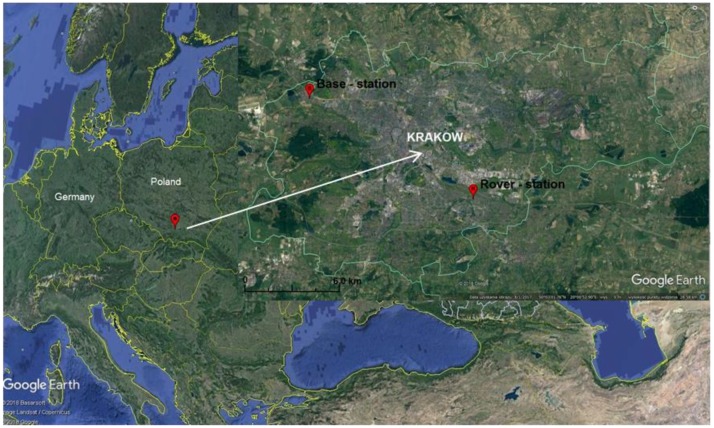
Location of the research facility (Base-station and Rover-station). Source: own calculation based on GoogleEarth.

The location of the base station relative to the rover receiver is shown in Figure 4. The distance of the base station from the rover receiver that performed RTK measurements was approximately 13.9 km. Both the base station and the rover receiver were located in places with favorable conditions for satellite measurements. It was assumed that the reference station and the rover receiver received the same signals from the Galileo satellites (E1, E5-A, E5-B, E5-AltBOC).

## 3. Methodology and Results for Testing the Accuracy of Measurements

The research involved a detailed analysis of 36 independent time series of coordinates, with the sampling interval of 10 s. The series were selected in such a way that the number (*n*) of measurements ranged from 160 to 360. A classic approach was adopted assuming that the average approaches the true value. In each series the following were determined:

I—systematic measurement error for each coordinate by formulae, respectively:

(1) $$\delta_{x}^{j}=\frac{\sum_{i=1}^{i=n}\Delta x_{i}}{n},\;\delta_{y}^{j}=\frac{\sum_{i=1}^{i=n}\Delta y_{i}}{n},\;\delta_{h}^{j}=\frac{\sum_{i=1}^{i=n}\Delta h_{i}}{n}$$

where: $\Delta x_{i}=x_{i}-x_{k}$, $\Delta y_{i}=y_{i}-y_{k}$, $\Delta h_{i}=h_{i}-h_{k}$—absolute errors of the *i*-th measurement, for coordinates: north (*x*), east (*y*), height (*h*), respectively; $\left(x_{i},y_{i},h_{i}\right)$—coordinates of the measured control point based on the *i*-th measurement, $\left(x_{k},y_{k},h_{k}\right)$—reference coordinates, (true) of the checkpoint, j—number of the series, n—length of the series.

To show the variability, the basic numerical characteristics of the systematic errors of measurement and the length of confidence intervals were determined for average values, estimated at a confidence level of 0.95 (Table 9 and Table 10).

If Galileo constellation is not complete, some discontinuities in observations occur. Then the solutions can be made only in selected “time windows”, when the number of observed satellites exceeds 4. The breaks in measurements cause that there is no continuity of observation and therefore systematic errors occur which depend mostly on the length of the breaks. However, in this paper only such measurement series were chosen for the analysis, in which there were no breaks.

II—average error of a single coordinate measurement according to the formulae, respectively:

(2) $$m_{\Delta x}=\sqrt{\frac{\sum_{i=1}^{i=n}\Delta x_{i}{}^{2}}{n}},\;m_{\Delta y}=\sqrt{\frac{\sum_{i=1}^{i=n}\Delta y_{i}{}^{2}}{n}},\;m_{\Delta h}=\sqrt{\frac{\sum_{i=1}^{i=n}\Delta h_{i}{}^{2}}{n}}$$

or:

(3) $$m_{v_{x}}=\sqrt{\frac{\sum_{i=1}^{i=n}v_{x_{i}}^{2}}{n}},\;m_{v_{y}}=\sqrt{\frac{\sum_{i=1}^{i=n}v_{y_{i}}^{2}}{n}},\;m_{v_{x}}=\sqrt{\frac{\sum_{i=1}^{i=n}v_{x_{i}}^{2}}{n}}$$

where: $\left(v_{x_{i}},v_{y_{i}},v_{h_{i}}\right):=\left(\Delta x_{i}-\delta_{x}^{j},\Delta y_{i}-\delta_{y}^{j},\Delta h_{i}-\delta_{h}^{j}\right)$—educed errors of the *i*-th measurement of the corresponding coordinate.

Table 11 Shows the average errors of a single measurement for each series in two variants: A—without taking into account the reduction of absolute errors, B—after taking into account the reduction of absolute errors.

Table 12 summarizes the basic numerical characteristics of the obtained average errors of a single measurement.

## 4. Discussion and Applications

The paper shows that despite the incomplete operability of the Galileo system, accurate, real-time geodetic measurements can be made using only Galileo satellites signals. The disadvantage of the system is availability of solutions in specific time windows. For the needs of this research, experimental test measurements were successfully performed at a static control point within 6 days at the end of 2017 and beginning of 2018. Three basic problems were examined in this paper, important from the point of view of a surveyor performing geodetic measurements for cadastral works or engineering geodesy tasks. The first one, covering the availability and continuity of positioning. The second one concerning the convergence time needed to achieve the measurement error not bigger than 0.05 m. The third one regarding accuracy of the point determination. As a measure of accuracy, the average error of determining a single point using the RTK was adopted. A three-stage study has shown that the current constellation, 14 fully operational satellites, provides in Krakow (Polska) in some selected periods of the day the possibility of performing precise RTK measurements using signals from Galileo satellites only. The first stage of research on the availability of RTK measurements was as follows (Table 1, Table 2, Table 3, Table 4, Table 5, Table 6 and Table 7):

I 362 DOY 2017—there were five observation windows with a total duration of 4 h 30 min
  - the minimum observation window lasted 20 min,
  - the maximum observation window lasted 2 h 30 min,
  - the average length of the observation window was 54 min,
II 363 DOY 2017—there were three observation windows with a total duration of 7 h 40 min
  - the minimum observation window lasted 10 min,
  - the maximum observation window lasted 3 h 50 min,
  - the average length of the observation window was 2 h 33 min,
III 364 DOY 2017—there were seven observation windows with a total duration of 10 h 20 min
  - the minimum observation window lasted 10 min,
  - the maximum observation window lasted 3 h 50 min,
  - the average length of the observation window was 1 h 28 min,
IV 365 DOY 2017—there were six observation windows with a total duration of 7 h 30 min
  - the minimum observation window lasted 30 min,
  - the maximum observation window lasted 3 h 40 min,
  - the average length of the observation window was 1 h 15 min,
V 001 DOY 2018—there were three observation windows with a total duration of 7 h 10 min
  - the minimum observation window lasted 30 min,
  - the maximum observation window lasted 3 h 30 min,
  - the average length of the observation window was 2 h 23 min,
VI 002 DOY 2018—there were ten observation windows with a total duration of 11 h 50 min
  - the minimum observation window lasted 10 min,
  - the maximum observation window lasted 5 h 00 min,
  - the average length of the observation window was 1 h 11 min,

The second stage of the research involved determination of the convergence time for the given accuracy (≤0.05 m) using Galileo satellites only. As it was shown in Table 8, 46% of the performed initializations did not exceed 10 s, while for over 63% it was shorter than one minute. Only in one of the thirty analyzed cases, the convergence time was longer than 10 min. One of the reasons in this case may be the high PDOP value. However, if we take into account the fact that the constellation of Galileo satellites was not complete during the test measurements and only 14 satellites could be used, this result should be considered positive and very promising in the future. Because the Galileo constellation is scheduled to reach the target number of operational satellites over the next 3 years, and then continuity of measurements will be assured 24 h a day. Moreover, during the tests, a higher power of observed Galileo signals in comparison to GPS signals was found.

The third stage included a detailed analysis of the accuracy of the RTK measurements. For this purpose, 36 independent RTK measurements series were made 10 s interval, at the cut horizontal angle of 10°. The duration of the series ranged from 30–60 min. In individual series, 160 to 360 measurements were registered.

The absolute (true) errors of time series of coordinates of the same control point were determined in the first place. Then, on their basis, the systematic errors of measurements were determined empirically (formula (1)) together with the length of confidence intervals (d/2) at a confidence level of 0.95 for each measurement series and individual coordinates (Table 9). It was shown that systematic errors are contained in the intervals: $\delta_{x}^{j}\in \left[-0.0171;0.0238\right]$ m, $\delta_{y}^{j}\in \left[0.0059;0.0306\right]$ m, $\delta_{h}^{j}\in \left[-0.0068;0.0330\right]$ m.

Next, average errors of a single measurement were determined (Table 11) in two variants (Formulas (2) and (3)), the first one did not take into account the reduction of absolute errors with the value of systematic errors, while the second one eliminated systematic errors from the observations. It was shown that the elimination of systematic errors significantly improves the accuracy of the average errors of a single measurement. The systematic errors depend on the quality of the constellation of satellites (PDOP) and from a specific set of satellites that is involved in the determination of the position.

It was shown (Table 12) that the average errors of a single RTK measurement based on a Galileo navigation system only are, respectively: for the northern coordinate (*x*): $m_{\Delta x}=\pm 0.0151$ *m*, for the eastern coordinate (*y*): $m_{\Delta y}=\pm 0.0291$ *m*, for the height (*h*): $m_{\Delta h}=\pm 0.0294$ *m*.

The elimination of systematic errors improves the accuracy of the average error of a single RTK measurement for the northern coordinate (*x*)—of about 9%, for the eastern coordinate (*y*)—of about 68% and for the height (*h*)—of about 23%.

Due to the well-known features and advantages of global navigation systems, the results presented above can be considered as the reliable for a wider area of Poland and even for Central and Eastern Europe. In order to confirm and increase their credibility, this type of research is needed elsewhere in Europe and in the world. However, it depends not only on the availability of Galileo satellites, but also on the ground infrastructure enabling corrections to RTK measurements.

## 5. Conclusions

The main advantage of the global navigation Galileo system is that it is civilian and not military. There is a hope that it will not be disabled for civilian users during military or political conflicts as it happened in the case of GPS and GLONASS systems. However, Galileo signal is more powerful than the GPS ones despite of 3 thousand kilometers bigger distance to its satellites than for GPS satellites.

The detailed studies have shown that although the Galileo system is not yet fully operational, currently it can be used to perform geodetic measurements in the real time with the centimeter accuracy. However, this is limited to the certain strictly defined time windows to which the user must currently adapt. For the place of performed tests, it was demonstrated that within 6 consecutive days the constellation of Galileo satellites made it possible to perform measurements for a time period from 18.75% to 49.31% of a day. And the size of the time window in which measurements were possible ranged from 10 min to a maximum of 5 h. The convergence time of the receiver up to the specified accuracy of ≤0.05 m for the individual measuring sessions varied and ranged from 10 s to 10 min 10 s. The systematic measurement errors for horizontal coordinates reached the range (−0.017 m–+0.031 m) and for the height (−0.007 m–+0.033 m). They can be caused by the discontinuities in observations. However, due to the fact that the Galileo system is just being built, it is not possible to eliminate such systematic errors. In various places on the globe, these errors can reach different values, e.g., depending on the discontinuity time span. Studies have shown that the Galileo system may already be useful in the real time positioning.

## Figures and Tables

**Table 1:** Galileo satellites availability for the Day of Year: 362, 2017.

Local Time: 2017-12-28 00:00:00–2017-12-29 00:00:00 (UTC + 2) Cutoff 10°				
Initial Time	End Time	Number of Satellites	Visible Satellites	Period of Time
00:00:00	11:20:00	2–4	E < 5	11:20:00
11:20:00	12:30:00	5	E02, E07, E08, E26, E30	01:10:00
12:30:00	13:00:00	6	E02, E07, E08, E12, E26, E30	00:30:00
13:00:00	13:10:00	7	E02, E07, E08, E12, E19, E26, E30	00:10:00
13:10:00	13:50:00	5	E02, E07, E12, E19, E30	00:40:00
13:50:00	19:30:00	2–4	E < 5	05:40:00
19:30:00	19:50:00	5	E01, E04, E09, E19, E26	00:20:00
19:50:00	20:50:00	4	E < 5	01:00:00
20:50:00	21:20:00	5	E01, E04, E05, E09, E26	00:30:00
21:20:00	21:40:00	4	E < 5	00:20:00
21:40:00	22:20:00	5	E01, E05, E09, E24, E26	00:40:00
22:20:00	22:30:00	4	E < 5	00:10:00
22:30:00	23:00:00	5	E01, E05, E12, E24, E26	00:30:00
23:00:00	00:00:00	4	E < 5	02:00:00

**Table 2:** Galileo satellites availability for the Day of Year: 363, 2017.

Local Time: 2017-12-29 00:00:00–2017-12-30 00:00:00 (UTC + 2) Cutoff 10°				
Initial Time	End Time	Number of Satellites	Visible Satellites	Period of Time
00:00:00	02:40:00	2–4	E < 5	02:40:00
02:40:00	03:40:00	5	E02, E11, E12, E19, E24	01:00:00
03:40:00	04:10:00	6	E02, E04, E11, E12, E19, E24	00:30:00
04:10:00	05:00:00	5	E02, E04, E11, E12, E19	00:50:00
05:00:00	05:50:00	7	E02, E04, E09, E11, E12, E19, E30	00:50:00
05:50:00	06:10:00	6	E02, E04, E09, E11, E19, E30	00:20:00
06:10:00	06:30:00	5	E02, E04, E09, E11, E30	00:20:00
06:30:00	06:50:00	4	E < 5	00:20:00
06:50:00	07:00:00	5	E04, E05, E09, E11, E30	00:10:00
07:00:00	13:10:00	2–4	E < 5	06:10:00
13:10:00	13:20:00	5	E02, E03, E05, E08, E24	00:10:00
13:20:00	14:10:00	6	E02, E03, E05, E08, E24, E26	00:50:00
14:10:00	14:20:00	5	E02, E03, E08, E24, E26	00:10:00
14:20:00	14:40:00	6	E02, E03, E07 E08, E24, E26	00:20:00
14:40:00	15:00:00	5	E02, E03, E07 E08, E26	00:20:00
15:00:00	16:50:00	5	E02, E03, E07 E08, E30	01:50:00
16:50:00	00:00:00	1–4	E < 5	07:10:00

**Table 3:** Galileo satellites availability for the Day of Year: 364, 2017.

Local Time: 2017-12-30 00:00:00–2017-12-31 00:00:00 (UTC + 2) Cutoff 10°				
Initial Time	End Time	Number of Satellites	Visible Satellites	Period of Time
00:00:00	03:30:00	2–4	E < 5	03:30:00
03:30:00	04:20:00	5	E01, E07, E08, E24, E26	00:50:00
04:20:00	04:40:00	6	E01, E07, E08, E12, E24, E26	00:20:00
04:40:00	04:50:00	5	E07, E08, E12, E24, E26	00:10:00
04:50:00	05:30:00	4	E < 5	00:40:00
05:30:00	06:40:00	5	E07, E12, E19, E24, E26	01:10:00
06:40:00	09:50:00	2–4	E < 5	03:10:00
09:50:00	11:00:00	5	E04, E09, E11, E12, E19	01:10:00
11:00:00	11:10:00	6	E01, E04, E09, E11, E12, E19	00:10:00
11:10:00	12:10:00	5	E01, E04, E09, E11, E19	01:00:00
12:10:00	12:40:00	4	E < 5	00:30:00
12:40:00	12:50:00	5	E01, E04, E05, E09, E11	00:10:00
12:50:00	13:50:00	4	E < 5	01:00:00
13:50:00	15:10:00	5	E01, E04, E05, E09, E24	01:20:00
15:10:00	15:20:00	4	E < 5	00:10:00
15:20:00	15:30:00	5	E01, E03, E05, E09, E24	00:10:00
15:30:00	18:10:00	3–4	E < 5	02:40:00
18:10:00	18:20:00	5	E02, E03, E05, E08, E24	00:10:00
18:20:00	19:10:00	6	E02, E03, E05, E08, E12, E24	00:50:00
19:10:00	19:30:00	7	E02, E03, E05, E08, E11, E12, E24	00:20:00
19:30:00	20:10:00	6	E02, E03, E08, E11, E12, E24	00:40:00
20:10:00	20:50:00	7	E02, E03, E07, E08, E11, E12, E24	00:40:00
20:50:00	21:00:00	6	E02, E03, E07, E08, E11, E12	00:10:00
21:00:00	21:30:00	6	E02, E07, E08, E11, E12, E30	00:30:00
21:30:00	22:00:00	5	E02, E07, E08, E11, E30	00:30:00
22:00:00	00:00:00	2–4	E < 5	02:00:00

**Table 4:** Galileo satellites availability for the Day of Year: 365, 2017.

Local Time: 2017-12-31 00:00:00–2018-01-01 00:00:00 (UTC + 2) Cutoff 10°				
Initial Time	End Time	Number of Satellites	Visible Satellites	Period of Time
00:00:00	05:20:00	2	E < 5	05:20:00
05:20:00	05:50:00	5	E01, E03, E05, E08, E26	00:30:00
05:50:00	06:20:00	4	E < 5	00:30:00
06:20:00	06:50:00	5	E01, E03, E07, E08, E26	00:30:00
06:50:00	10:20:00	4	E < 5	03:30:00
10:20:00	10:30:00	5	E02, E07, E08, E12, E26	00:10:00
10:30:00	10:40:00	6	E02, E07, E08, E12, E26, E30	00:10:00
10:40:00	10:50:00	7	E02, E07, E08, E12, E19, E26, E30	00:10:00
10:50:00	11:50:00	6	E02, E07, E12, E19, E26, E30	01:00:00
11:50:00	12:40:00	4	E < 5	00:50:00
12:40:00	13:30:00	5	E07, E11, E12, E19, E30	00:50:00
13:30:00	13:40:00	4	E < 5	00:10:00
13:40:00	14:10:00	5	E04, E11, E12, E19, E30	00:30:00
14:10:00	18:40:00	3	E < 5	04:30:00
18:40:00	19:10:00	5	E01, E04, E05, E09, E26	00:30:00
19:10:00	20:00:00	6	E01, E04, E05, E09, E24, E26	00:50:00
20:00:00	21:00:00	5	E01, E05, E09, E24, E26	01:00:00
21:00:00	21:10:00	6	E01, E05, E09, E12, E24, E26	00:10:00
21:10:00	21:20:00	7	E01, E03, E05, E09, E12, E24, E26	00:10:00
21:20:00	21:50:00	6	E01, E03, E05, E12, E24, E26	00:30:00
21:50:00	22:20:00	5	E03, E05, E12, E24, E26	00:30:00
22:20:00	00:00:00	3	E < 5	01:40:00

**Table 5:** Galileo satellites availability for the Day of Year: 001, 2018.

Local Time: 2018-01-01 00:00:00–2018-01-02 00:00:00 (UTC+2) Cutoff 10°				
Initial Time	End Time	Number of Satellites	Visible Satellites	Period of Time
00:00:00	02:00:00	3–4	E < 5	02:00:00
02:00:00	02:30:00	5	E02, E11, E12, E19, E24	00:30:00
02:30:00	02:40:00	4	E < 5	00:10:00
02:40:00	02:50:00	5	E02, E04, E11, E12, E19	00:10:00
02:50:00	03:30:00	6	E02, E04, E11, E12, E19, E30	00:40:00
03:30:00	03:40:00	5	E02, E04, E11, E19, E30	00:10:00
03:40:00	04:00:00	6	E02, E04, E09, E11, E19, E30	00:20:00
04:00:00	05:10:00	5	E02, E04, E09, E11, E30	01:10:00
05:10:00	05:30:00	6	E02, E04, E05, E09, E11, E30	00:20:00
05:30:00	06:10:00	5	E04, E05, E09, E11, E30	00:40:00
06:10:00	11:20:00	2–4	E < 5	05:10:00
11:20:00	11:50:00	5	E03, E05, E08, E24, E26	00:30:00
11:50:00	12:00:00	5	E02, E03, E08, E24, E26	00:10:00
12:00:00	12:40:00	6	E02, E03, E07, E08, E24, E26	00:40:00
12:40:00	13:20:00	5	E02, E03, E07, E08, E26	00:40:00
13:20:00	14:30:00	6	E02, E03, E07, E08, E26, E30	01:10:00
14:30:00	00:00:00	2–4	E < 5	09:30:00

**Table 6:** Galileo satellites availability for the Day of Year: 002, 2018.

Local Time: 2018-01-02 00:00:00–2018-01-03 00:00:00 (UTC + 2) Cutoff 10°				
Initial Time	End Time	Number of Satellites	Visible Satellites	Period of Time
00:00:00	02:00:00	2–4	E < 5	02:00:00
02:00:00	02:10:00	5	E01, E08, E12, E24, E26	00:10:00
02:10:00	02:30:00	6	E01, E07, E08, E12, E24, E26	00:20:00
02:30:00	03:10:00	5	E01, E07, E12, E24, E26	00:40:00
03:10:00	04:00:00	4	E < 5	00:50:00
04:00:00	04:30:00	5	E07, E12, E19, E24, E26	00:30:00
04:30:00	04:40:00	4	E < 5	00:10:00
04:40:00	05:10:00	5	E07, E11, E12, E19, E24	00:30:00
05:10:00	05:40:00	4	E < 5	00:30:00
05:40:00	05:50:00	5	E04, E11, E12, E19, E24	00:10:00
05:50:00	06:10:00	4	E < 5	00:20:00
06:10:00	06:50:00	5	E02, E04, E11, E12, E19	00:40:00
06:50:00	07:50:00	4	E < 5	01:00:00
07:50:00	09:30:00	5	E04, E09, E11, E12, E19	01:40:00
09:30:00	10:10:00	3–4	E < 5	00:40:00
10:10:00	11:20:00	5	E01, E04, E05, E09, E11	01:10:00
11:20:00	12:20:00	4	E < 5	01:00:00
12:20:00	12:50:00	5	E01, E04, E05, E09, E24	00:30:00
12:50:00	13:00:00	3–4	E < 5	00:10:00
13:00:00	13:30:00	5	E01, E03, E05, E09, E24	00:30:00
13:30:00	16:00:00	3–4	E < 5	02:30:00
16:00:00	17:50:00	5	E02, E03, E05, E08, E24	01:50:00
17:50:00	18:10:00	5	E02, E03, E08, E12, E24	00:20:00
18:10:00	18:20:00	6	E02, E03, E07, E08, E12, E24	00:10:00
18:20:00	18:30:00	6	E02, E03, E07, E08, E11, E12	00:10:00
18:30:00	19:30:00	7	E02, E03, E07, E08, E11, E12, E30	01:00:00
19:30:00	19:40:00	6	E02, E03, E07, E08, E11, E30	00:10:00
19:40:00	21:00:00	5	E02, E07, E08, E11, E30	01:20:00
21:00:00	00:00:00	2–4	E < 5	03:00:00

**Table 7:** Visibility time of at least 5 Galileo satellites, within 24 h, at the elevation >10°.

Day of Year (DOY)	362 DOY 2017	363 DOY 2017	364 DOY 2017	365 DOY 2017	001 DOY 2018	002 DOY 2018
The number of Galileo satellites, 5 and more	18.75%/day	31.94%/day	43.05%/day	31.25%/day	29.86%/day	49.31%/day

**Table 8:** The list of obtained convergence times.

No.	Convergence Times h:min:s	Value of PDOP		Number of Observed Satellites	
		At the Start	After Reaching the Assumed Accuracy	At the Start	After Reaching the Assumed Accuracy
1	00:00:10	1.87	1.87	5	5
2	00:00:10	1.92	1.92	7	7
3	00:00:10	1.93	1.93	5	5
4	00:00:10	1.98	1.98	7	7
5	00:00:10	2.04	2.04	6	6
6	00:00:10	2.24	2.24	6	6
7	00:00:10	2.47	2.48	5	5
8	00:00:10	2.55	2.55	5	5
9	00:00:10	2.83	2.83	5	5
10	00:00:10	2.88	2.88	5	5
11	00:00:10	2.94	2.95	5	5
12	00:00:10	2.97	2.97	5	5
13	00:00:10	2.99	2.81	5	5
14	00:00:10	2.99	2.99	5	5
15	00:00:20	3.54	3.54	5	5
16	00:00:20	4.81	4.78	5	5
17	00:00:30	3.73	3.74	6	6
18	00:00:40	4.12	4.11	5	5
19	00:00:50	3.26	3.26	5	5
20	00:01:20	3.04	3.03	5	5
21	00:02:30	6.01	2.74	5	6
22	00:02:50	3.12	3.13	5	5
23	00:03:40	5.84	3.04	6	7
24	00:04:00	3.68	2.08	5	6
25	00:05:20	6.21	3.52	5	5
26	00:05:50	3.61	3.59	5	5
27	00:06:02	2.82	2.52	5	5
28	00:08:40	2.99	2.85	5	5
29	00:09:20	3.99	3.74	6	6
30	00:10:10	3.96	3.91	5	6

Source: Own elaboration based on performed experimental tests.

**Table 9:** Systematic errors of measurements together with the length of confidence intervals (d/2) at the confidence level of 0.95 and the minimum (PDOPmin) and maximum (PDOPmax) values of PDOP parameters for each series.

No of Series *j*	$\delta_{x}^{j}\;\pm \;d/2$		$\delta_{y}^{j}\;\pm \;d/2$		$\delta_{h}^{j}\;\pm \;d/2$		PDOP_min_	PDOP_max_
	[m]		[m]		[m]			
1	−0.0050	±0.0011	0.0281	±0.0011	0.0253	±0.0014	2.064	2.563
2	−0.0014	±0.0019	0.0263	±0.0009	0.0154	±0.0023	2.475	3.577
3	0.0033	±0.0018	0.0248	±0.0018	0.0132	±0.0109	3.920	4.813
4	0.0130	±0.0010	0.0175	±0.0009	0.0288	±0.0025	2.162	2.370
5	0.0042	±0.0010	0.0280	±0.0006	0.0287	±0.0013	2.243	3.150
6	−0.0009	±0.0010	0.0194	±0.0007	0.0049	±0.0012	1.838	2.990
7	0.0029	±0.0010	0.0160	±0.0009	0.0189	±0.0024	1.913	3.068
8	0.0040	±0.0011	0.0218	±0.0014	0.0240	±0.0045	3.494	4.124
9	−0.0171	±0.0051	0.0220	±0.0021	0.0119	±0.0030	3.539	3.966
10	−0.0169	±0.0077	0.0237	±0.0021	0.0173	±0.0037	3.590	3.606
11	0.0015	±0.0008	0.0289	±0.0006	0.0124	±0.0013	1.873	2.826
12	−0.0022	±0.0010	0.0267	±0.0009	0.0057	±0.0018	2.033	4.324
13	−0.0026	±0.0012	0.0281	±0.0010	0.0176	±0.0013	3.064	3.242
14	−0.0085	±0.0009	0.0254	±0.0008	0.0299	±0.0030	3.079	3.254
15	−0.0009	±0.0009	0.0274	±0.0012	0.0158	±0.0019	1.966	5.969
16	0.0310	±0.0026	0.0059	±0.0069	0.0203	±0.0009	1.912	3.002
17	0.0033	±0.0068	0.0232	±0.0036	−0.0062	±0.0034	1.687	2.625
18	0.0158	±0.0063	0.0289	±0.0006	−0.0068	±0.0029	2.038	4.126
19	−0.0041	±0.0008	0.0282	±0.0007	0.0093	±0.0014	2.759	3.017
20	−0.0016	±0.0012	0.0306	±0.0013	0.0040	±0.0019	1.867	3.326
21	0.0010	±0.0009	0.0290	±0.0005	0.0005	±0.0017	2.324	3.242
22	−0.0011	±0.0008	0.0283	±0.0006	0.0185	±0.0036	3.082	3.933
23	−0.0012	±0.0008	0.0273	±0.0007	−0.0003	±0.0025	1.853	5.842
24	0.0065	±0.0020	0.0256	±0.0017	0.0092	±0.0036	2.070	3.099
25	0.0025	±0.0012	0.0193	±0.0020	−0.0029	±0.0024	2.082	3.685
26	0.0042	±0.0010	0.0283	±0.0006	0.0081	±0.0033	2.480	5.821
27	0.0004	±0.0017	0.0250	±0.0009	0.0077	±0.0019	2.067	3.681
28	0.0238	±0.0035	0.0116	±0.0032	0.0306	±0.0031	2.669	3.021
29	0.0019	±0.0008	0.0279	±0.0006	0.0178	±0.0022	2.120	3.059
30	−0.0080	±0.0016	0.0270	±0.0005	0.0253	±0.0080	3.065	6.127
31	0.0023	±0.0014	0.0285	±0.0006	0.0222	±0.0051	2.388	5.834
32	0.0033	±0.0016	0.0215	±0.0023	0.0166	±0.0038	1.853	1.873
33	−0.0035	±0.0009	0.0296	±0.0008	0.0151	±0.0019	2.808	2.987
34	0.0006	±0.0006	0.0240	±0.0007	0.0087	±0.0019	2.882	3.703
35	−0.0001	±0.0011	0.0302	±0.0020	0.0330	±0.0063	1.873	6.579
36	0.0017	±0.0015	0.0236	±0.0015	0.0065	±0.0020	1.916	1.954

**Table 10:** Basic statistics for systematic errors of measurements together with the length of confidence intervals (d/2) at the confidence level of 0.95 and the minimum (PDOPmin) and maximum (PDOPmax) values of PDOP parameters for each series.

Values of Statistics	δ_x_ ± d/2		δ_y_ ± d/2		δ_H_ ± d/2		PDOP_min_	PDOP_max_
	[m]		[m]		[m]			
***min***	−0.0171	±0.0051	0.0059	±0.0005	−0.0068	±0.0012	1.687	1.873
***max***	0.0238	±0.0035	0.0306	±0.0046	0.0330	±0.0109	3.920	6.579
***AV***	0.0011	±0.0018	0.0247	±0.0012	0.0141	±0.0030	2.418	3.733
***SD***	0.0081	±0.0018	0.0054	±0.0008	0.0105	±0.0020	0.601	1.208
***V***	717.1%	98.4%	21.8%	±67.9%	74.6%	±67.0%	24.9%	32.4%

**Table 11:** List of average errors of a single measurement, respectively: A—without taking into account the reduction of absolute errors, B—after taking into account the reduction of absolute errors respectively.

No of Series *j*	A			B			Number of Measurements
	$\pm m_{\Delta x}$	$\pm m_{\Delta y}$	$\pm m_{\Delta h}$	$\pm m_{v_{x}}$	$\pm m_{v_{y}}$	$\pm m_{v_{h}}$	
	[m]	[m]	[m]	[m]	[m]	[m]	
1	0.0092	0.0292	0.0272	0.0078	0.0080	0.0100	197
2	0.0144	0.0272	0.0233	0.0143	0.0068	0.0175	231
3	0.0076	0.0257	0.0444	0.0069	0.0070	0.0424	160
4	0.0160	0.0195	0.0367	0.0093	0.0086	0.0229	326
5	0.0103	0.0285	0.0315	0.0094	0.0058	0.0129	361
6	0.0098	0.0205	0.0126	0.0097	0.0066	0.0116	361
7	0.0076	0.0173	0.0253	0.0070	0.0067	0.0167	190
8	0.0077	0.0235	0.0369	0.0066	0.0088	0.0281	178
9	0.0454	0.0280	0.0272	0.0420	0.0174	0.0245	261
10	0.0287	0.0245	0.0206	0.0232	0.0063	0.0111	163
11	0.0083	0.0294	0.0178	0.0082	0.0058	0.0128	361
12	0.0100	0.0282	0.0181	0.0097	0.0090	0.0172	361
13	0.0118	0.0297	0.0214	0.0118	0.0098	0.0122	361
14	0.0109	0.0261	0.0386	0.0069	0.0064	0.0244	250
15	0.0065	0.0287	0.0206	0.0064	0.0083	0.0132	182
16	0.0094	0.0847	0.0581	0.0660	0.0440	0.0326	361
17	0.0728	0.0443	0.0383	0.0118	0.0114	0.0159	361
18	0.0600	0.0294	0.0275	0.0579	0.0055	0.0266	324
19	0.0087	0.0291	0.0169	0.0076	0.0071	0.0141	380
20	0.0097	0.0326	0.0165	0.0095	0.0110	0.0160	264
21	0.0087	0.0294	0.0162	0.0086	0.0052	0.0162	361
22	0.0057	0.0286	0.0303	0.0055	0.0042	0.0240	169
23	0.0076	0.0281	0.0239	0.0075	0.0065	0.0239	361
24	0.0189	0.0297	0.0325	0.0178	0.0150	0.0312	293
25	0.0102	0.0251	0.0195	0.0099	0.0162	0.0193	258
26	0.0091	0.0287	0.0279	0.0080	0.0049	0.0267	251
27	0.0162	0.0266	0.0202	0.0162	0.0091	0.0186	361
28	0.0301	0.0206	0.0347	0.0184	0.0170	0.0163	160
29	0.0082	0.0286	0.0277	0.0080	0.0061	0.0212	361
30	0.0176	0.0274	0.0815	0.0157	0.0050	0.0774	361
31	0.0110	0.0289	0.0457	0.0108	0.0049	0.0400	237
32	0.0064	0.0230	0.0212	0.0056	0.0082	0.0132	164
33	0.0073	0.0301	0.0199	0.0064	0.0056	0.0129	182
34	0.0059	0.0250	0.0205	0.0059	0.0069	0.0186	361
35	0.0110	0.0359	0.0691	0.0110	0.0195	0.0607	361
36	0.0051	0.0242	0.0092	0.0048	0.0050	0.0065	162

**Table 12:** List of basic measurement statistics, respectively: A—without taking into account the reduction of absolute errors, B—after taking into account the reduction of absolute errors respectively.

Statistics	A			B		
	$\pm m_{\Delta x}$	$\pm m_{\Delta y}$	$\pm m_{\Delta h}$	$\pm m_{v_{x}}$	$\pm m_{v_{y}}$	$\pm m_{v_{h}}$
	[m]	[m]	[m]	[m]	[m]	[m]
***min***	0.0051	0.0173	0.0092	0.0048	0.0042	0.0065
***max***	0.0728	0.0847	0.0815	0.0660	0.0440	0.0774
***AV***	0.0151	0.0291	0.0294	0.0137	0.0094	0.0225
***SD***	0.0150	0.0106	0.0152	0.0137	0.0071	0.0142
***V***	99.3%	36.5%	51.7%	100.1%	75.4%	62.9%

## References

[1] Li X., Ge M., Dai X., Ren X., Fritsche M., Wickert J., Schuh H. (2015). Accuracy and reliability of multi-GNSS real-time precise positioning: GPS, GLONASS, BeiDou, and Galileo. J. Geod..

[2] Li X., Dick G., Ge M., Heise S., Wickert J., Bender M. (2014). Real-time GPS sensing of atmospheric water vapor: Precise point positioning with orbit, clock, and phase delay corrections. Geophys. Res. Lett..

[3] Paziewski J., Wielgosz P. (2015). Accounting for Galileo–GPS inter-system biases in precise satellite positioning. J. Geod..

[4] Siejka Z. (2015). Multi-GNSS as a combination of GPS, GLONASS and BeiDou measurements carried out in real time. Artif. Satell..

[5] Gao W., Gao C., Pan S.A. (2016). Method of GPS/BDS/GLONASS combined RTK positioning for middle-long baseline with partial ambiguity resolution. Surv. Rev..

[6] Pan L., Cai C., Santerre R., Zhang X. (2017). Performance evaluation of single-frequency point positioning with GPS, GLONASS, BeiDou and Galileo. Surv. Rev..

[7] Guo F., Li X., Zhang X., Wang J. (2017). The contribution of Multi-GNSS Experiment (MGEX) to precise point positioning. Adv. Space Res..

[8] Montenbruck O., Steigenberger P., Prange L., Deng Z., Zhao Q., Perosanz F., Romero I., Noll C., Sturze A., Weber G. (2017). The Multi-GNSS Experiment (MGEX) of the International GNSS Service (IGS)-Achievements, prospects and challenges. Adv. Space Res..

[9] Rizos C., Janssen V., Roberts C., Grinter T. Precise Point Positioning: Is the Era of Differential GNSS Positioning Drawing to the End. Proceedings of the FIG Working Week 2012.

[10] Siejka Z. (2014). Verification of the usefulness of the trimble RTX extended satellite technology with the xfill function in the local network implementing RTK measurements. Artif. Satell..

[11] Pan L., Xiaohong Z., Fei G. (2017). Ambiguity resolved precise point positioning with GPS and BeiDou. J. Geod..

[12] Bakuła M. (2013). Study of Reliable Rapid and Ultrarapid Static GNSS Surveying for Determination of the Coordinates of Control Points in Obstructed Conditions. J. Surv. Eng..

[13] Dawidowicz K., Krzan G., Świątek K. Relative GPS/GLONASS coordinates determination in urban areas-accuracy analysis. Proceedings of the 15th SGEM GeoConferences.

[14] Dawidowicz K., Krzan G. (2014). Accuracy of single receiver static GNSS measurements under conditions of limited satellite availability. Surv. Rev..

[15] Wang L., Li Z., Ge M., Neitzel F., Wang Z., Yuan H. (2018). Validation and Assessment of Multi-GNSS Real-Time Precise Point Positioning in Simulated Kinematic Mode Using IGS Real-Time Service. Remote Sens..

[16] White Paper Trimble Survey Division Westminster (2012). Trimble Hd-Gnss Processing. https://community.trimble.com.

[17] Wang L., Li Z., Yuan H., Zhao J., Zhou K., Yuan C. (2016). Influence of the time-delay of correction for BDS and GPS combined real-time differential positioning. Electron. Lett..

